# Idiopathic eosinophilic pneumonia in children: the French experience

**DOI:** 10.1186/1750-1172-9-28

**Published:** 2014-02-20

**Authors:** Lisa Giovannini-Chami, Alice Hadchouel, Nadia Nathan, Francois Brémont, Jean-Christophe Dubus, Michael Fayon, Véronique Houdouin, Michèle Berlioz-Baudoin, Virginie Feret, Thierry Leblanc, Karine Morelle, Marc Albertini, Annick Clement, Jacques de Blic

**Affiliations:** 1 Pediatric Pulmonology and Allergy Department, Hôpitaux pédiatriques de Nice CHU-Lenval, Nice F-06200, France; 2 Université de Nice Sophia-Antipolis, Nice F-06000, France; 3 Pediatric Pulmonology and Allergy Department, AP-HP, Hôpital Necker Enfants Malades, Paris F-75015, France; 4 Université Paris Descartes-Paris 5, Paris F-75005, France; 5 Pediatric Pulmonology Department, AP-HP, Hôpital Trousseau, Paris F-75012, France; 6 Université Pierre et Marie Curie-Paris 6, Inserm, UMR S-U938, Paris F-75012, France; 7 Pediatric Pulmonology Department, Centre Hospitalier Universitaire de Toulouse, Toulouse, France; 8 Pediatric Pulmonology Department, Centre Hospitalier Universitaire de Marseille, Marseille, France; 9 Pediatric Pulmonology Department, Centre Hospitalier Universitaire de Bordeaux, Département de Pédiatrie, Centre d'Investigation Clinique (CIC 0005), F-33000 Bordeaux, France; 10 Pediatric Pulmonology Department, AP-HP, Hôpital Robert Debré, Paris F-75019, France; 11 Université paris Diderot VII, Paris F-75, France; 12 Pediatric Hematology Department, AP-HP, Hôpital Robert Debré, Paris F-75019, France

**Keywords:** Idiopathic chronic eosinophilic pneumonia, Idiopathic acute eosinophilic pneumonia, Child, Eosinophilic lung disease, Interstitial lung disease

## Abstract

**Background:**

Idiopathic eosinophilic pneumonia is extremely rare in children and adults. We present herein the first series describing the specificities of idiopathic chronic (ICEP) and acute (IAEP) eosinophilic pneumonia in children.

**Methods:**

We retrospectively analyzed all cases of ICEP and IAEP in children that were retrieved from French Reference Centers for rare pediatric lung diseases.

**Results:**

Five cases of pediatric ICEP were identified. Corticosteroid or immunosuppressive therapy dramatically improved the outcome in three cases. The remaining two cases had a persistent interstitial pattern with progressive development of cystic airspace lesions. Three cases of IAEP in adolescents were reported, with one requiring four days of extracorporeal membrane oxygenation.

**Conclusion:**

ICEP is a rare disease with a polymorphic clinical presentation in children. We identified patients with persistent interstitial patterns progressing to cystic airspace regions, for which the boundaries with idiopathic interstitial pneumonias are difficult to establish. We therefore propose a specific pediatric definition and classification algorithm. IAEP in children remains an inflammatory reaction of the lung to an acute toxic exposure, mainly tobacco, as in adults. International studies are required to comprehensively assess the various clinical forms of the disease as well as the appropriate therapeutic regimens.

## Introduction

Eosinophilic pneumonias (EP) [1] are diffuse, parenchymal lung diseases (DPLD) characterized by prominent eosinophil infiltration in the pulmonary interstitium and alveolar airspaces, exerting a major role in tissue injury. EP are divided into two groups: (i) secondary forms (seen mainly in parasitic infections, allergic bronchopulmonary aspergillosis (ABPA) and drug reactions, but also in other infections and neoplasia) and (ii) idiopathic forms (Eosinophilic Granulomatosis with Polyangiitis, idiopathic hypereosinophilic syndrome, idiopathic chronic eosinophilic pneumonia (ICEP) and idiopathic acute eosinophilic pneumonia (IAEP)). Idiopathic eosinophilic pneumonias are rare entities and pediatric case descriptions are scarce. They most likely do not represent one single, clearly defined disease entity but, rather, a common phenotype which shares pronounced eosinophilia but does not fulfill strict definitions or criteria. To date, no series of pediatric ICEP or IAEP have been reported. The objective of this multicenter retrospective study was to analyze cases of ICEP and IAEP in children.

## Patients and methods

### Recruitment

Three cases of ICEP were identified from the national, internet-linked database (created in 2008) for pediatric interstitial lung disease (ILD) of the National Reference Center for Rare Lung Diseases (Respirare®, http://www.respirare.fr) [2]. Data were available for 217 cases of ILD, with “eosinophilic lung diseases” being one of the less-frequent etiologies (5/217) [2].

As acute diseases are not included in the registry, cases of IAEP were retrieved by mailing to all members of the National Reference Center and to all members of the “Groupe Francophone de Réanimation et d’Urgences Pédiatriques”. This mailing process also permitted the identification of two supplementary cases of ICEP not yet registered and four other cases of eosinophilic lung diseases.

### Data collection

A detailed questionnaire regarding the initial presentation, investigations, elimination of differential diagnoses, treatment, outcome and follow-up was sent to referring physicians.

Inclusion criteria for ICEP and IAEP [3] were based on adult series (Table 1).

**Table 1:** **ICEP and IAEP inclusion criteria based on adults series**^**3**^

**Inclusion criteria for ICEP based on adults series**^**3**^	**Inclusion criteria for IAEP based on adults series**^**3**^
1) respiratory symptoms present> 2 weeks	1) acute onset with febrile respiratory symptoms (< 1 month, and especially < 7 days duration before medical examination)
2) diffuse pulmonary alveolar consolidation with air bronchogram and/or ground-glass opacities at chest imaging, especially with peripheral predominance	
	2) bilateral diffuse infiltrates on imaging
3) eosinophil count in bronchoalveolar lavage fluid (BALF) > 40% and/or peripheral blood eosinophilia > 1x10^9^cells/L	3) PaO2 in room air < 60 mmHg, or PaO2/FiO2 < 300 mmHg, or oxygen saturation (SpO2) in room air < 90%
4) absence of other known causes of eosinopilic lung disease*	4) lung eosinophilia with > 25% eosinophils in BALF
	5) absence of determined cause of acute eosinophilic pneumonia* (including infection or drug exposure) - recent onset of tobacco smoking or exposure to inhaled dusts may be present

Footnote: * We excluded patients with evidence of: 1) exposure to drugs inducing pulmonary eosinophilia, according to the Pneumotox website (http://www.pneumotox.com); 2) ABPA; 3) positive stool parasite test or positive serologic test for *Toxocara canis*, *Trichinella spiralis*, *Fasciola hepatica*, *Strongyloides stercoralis*, *Brugia malayi* or *Wuchereria bancrofti*; 4) vasculitis or malignancy at the time of diagnosis.

Six cases of EP were excluded because of the following: hypereosinophilic asthma (two cases); Eosinophilic Granulomatosis with Polyangiitis diagnosis (three cases); positive serologic test for *Wuchereria bancrofti* (one case).

### Statistical analysis

Due to the small number of patients in each group and the non normal distribution of values, we have presented median values with interquartile ranges.

## Results

### ICEP

#### ***Epidemiological data and medical history***

Five patients were identified (Table 2). There were four girls and one boy (sex ratio: 0.25). Mean age at diagnosis was 11.17 [6.83-13.92] years. All patients lived in metropolitan France at the time of diagnosis except for case 3 who lived in Martinique (a tropical overseas French territory in the Caribbean). Case 4 was born in Mali and case 1 had previously lived in Mayotte (another tropical overseas French territory in the Mozambique Channel). Three patients had a history of atopy, with three having asthma, two allergic rhinoconjunctivitis, two atopic dermatitis (one with a severe form), and one multiple food allergy (seafood, peanut, egg).

**Table 2:** ICEP patient characteristics at diagnosis

**Patient**	**Clinical presentation**							**Biology**												**Imagery**	**Spirometry**
	**Sex**	**Age at diagnosis (years)**	**Atopic history**	**Delay to diagnosis (days)**	**Respiratory symptoms**	**Respiratory exam**	**General symptoms**	**Initial blood Eo count/mm3**	**Maximal blood Eo count/mm3**	**Total IgE kU/L**	**Lymphocyte phenotype**	**T-cell clonality**	**ANA**	**BALF cellularity (cell/mm3)**	**BALF Eo %**	**BALF lymphocytes %**	**BALF neutrophils %**	**BALF macrophages %**	**Histology**	**CT scan**	
1	F	15.5	No	**60**	ED, RD, DC, T	DBS	yes	1510	**4600**	799	normal	No	1/1280	160,000	**28**	17	2	53	**nd**	**AO, GGO, N, IST, Ad, B, PE**	R, O, D
2	F	13.9	No	**60**	ED, RD, DC	W, DBS	yes	4601	**4600**	165	normal	No	_	390,000	**44**	6	4	46	**nd**	**AO, GGO, B, BV, Ad**	R, O, D
3	F	6.8	Yes	**120**	ED, PC	normal	no	1100	**1100**	>5000	normal	No	1/100	241,000	**21**	20	5	52	**Positive**	**dGGO, RN**	R
4	M	5	Yes	**90**	DC	W	no	33,580	**48,920**	150	2,2% CD3+ CD4- CD8- with TCR αβ	nd	_	450,000	**20**	5	2	73	**nd**	**pGGO, N, Ad**	R
5	F	11.2	Yes	**37**	ED, RD, DC, CP	H, P	yes	80	**1300**	1040	nd	Positive (TCR γ gene)	nd	112,000	**14**	16	43	27	**nd**	**dGGO, RN, Ad, PM**	nd

**Footnote:** F: female, M: male, nd: not determined, ED: exertional dyspnea, RD: rest dyspnea, DC: dry cough, T: chest tightness, PC: productive cough, CP: chest pain, DBS: decreased breath sound, W: wheezing, H: hypoxemia ,P: polypnea, Eo: eosinophils, ANA: antinuclear antibodies, BALF: broncho-alveolar lavage fluid, AO: alveolar opacities, GGO: ground-glass opacities, N: nodules, IST: interlobular septal thickening, Ad: adenopathy, B: bronchiectasis, PE: pleural effusions, BV: peri-broncho-vascular thickening, RN: reticulonodular syndrome, N, nodules, PM: pneumomediastinum, d: diffuse, p: patchy, , D: diffusion impairment, R: restriction, O: obstruction, in bold: diagnostic criteria.

#### ***Clinical data***

The median period between the onset of respiratory symptoms and diagnosis was 60 [60-90] days. Cough was the initial symptom in all cases (mostly dry cough: 4/5). Systemic symptoms always appeared secondarily, with a median period of 30 [25.5-30] days. Fever was present in only one patient (1/5) (case 1).

#### ***Radiological data***

**Figure 1:**
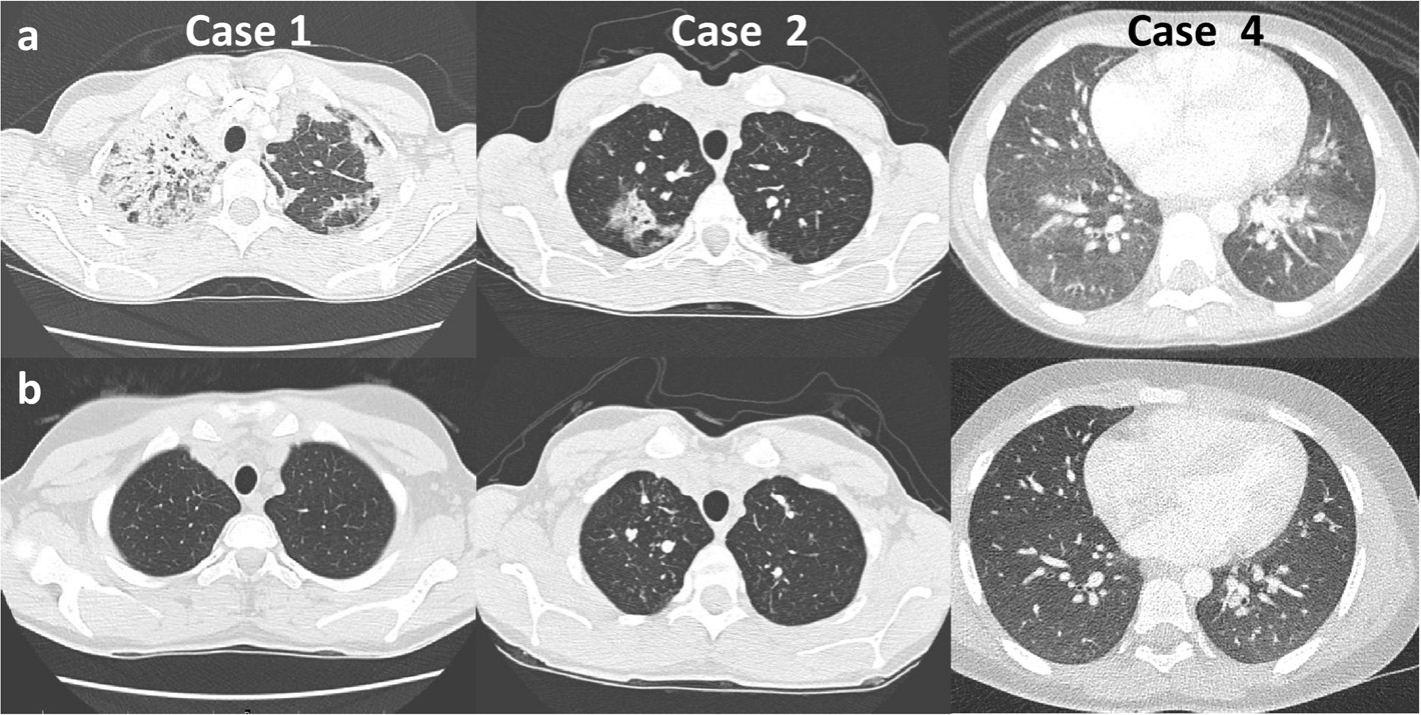
**HRCT scan in “dramatic improvement subset” ICEP patients. (a)** At diagnosis: in case 1, bilateral and peripheral, dense and fluffy alveolar opacities associated with ground-glass opacities predominantly in the right superior lobe; in case 2, peripheral, alveolar, dense and fluffy opacities with areas of ground-glass opacities predominantly in the right and also in the left apex; in case 4, patchy ground-glass opacities and nodules. **(b)** Last HRCT scan showing complete normalization.

**Figure 2:**
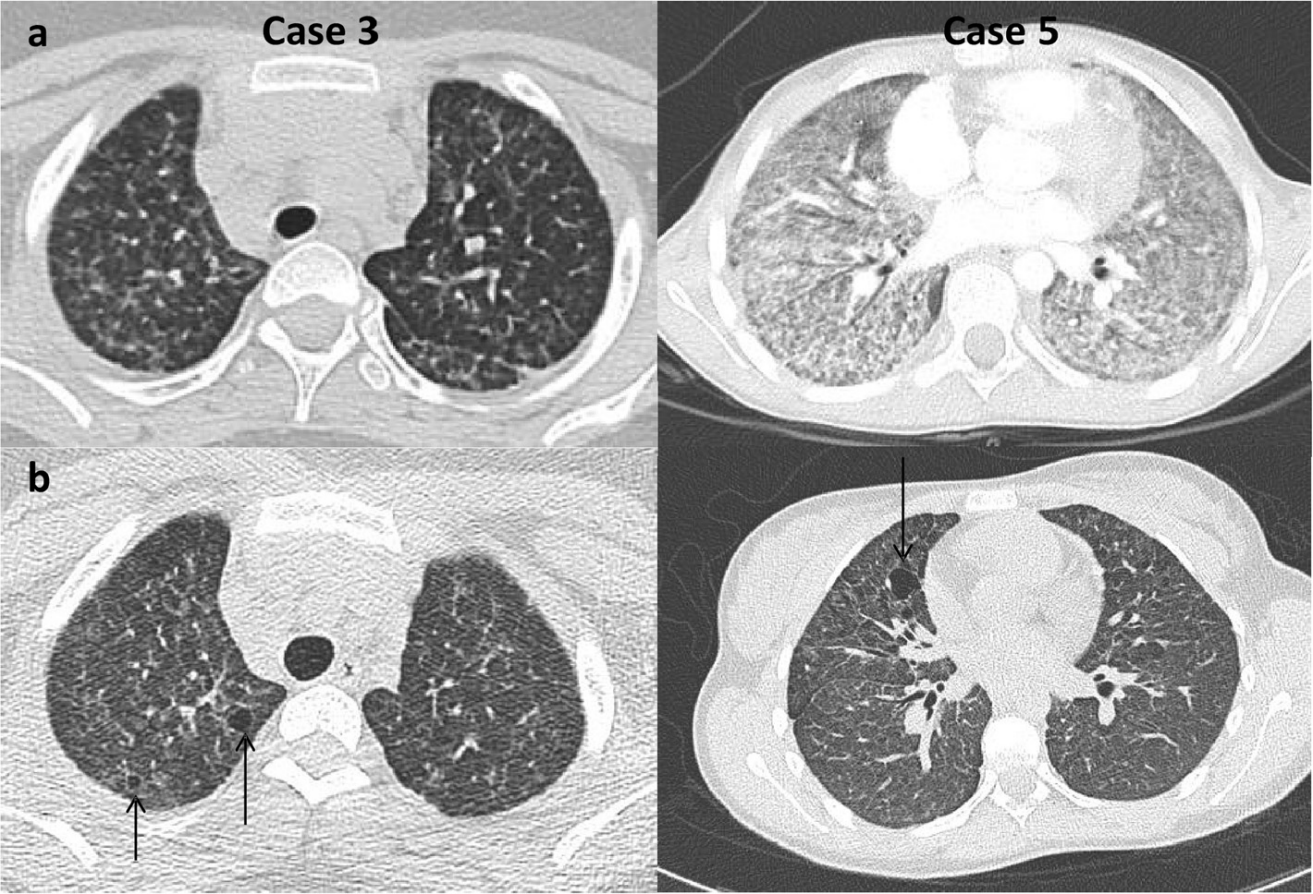
**HRCT scan in “persistent diffuse interstitial subset” ICEP patients. (a)** At diagnosis: in cases 3 and 5, diffuse ground glass opacities, reticulonodular syndrome. **(b)** Last HRCT scan showing diffuse ground-glass opacities with development of thin-walled cysts in cases 3 and 5.

Chest X-rays were abnormal in four patients, with two having dense infiltrates of the right upper lobe and two having bilateral, ill-defined, hazy increased densities. A high-resolution computed tomograph (HRCT) chest scan showed a typical adult-type ICEP presentation, including bilateral peripheral ground-glass and consolidation opacities (present in, respectively, 88 and 100% of adults with ICEP in a series of 80 readings) [4] in only two patients (cases 1 and 2) (Figure 1a). The three others presented only with interstitial opacities which were diffuse in cases 3 and 5 and patchy in the inferior lobes in case 4 (Figure 1a and 2a).

#### ***Biological data***

The blood eosinophil count on admission was > 1,000/mm^3^ in four patients, with a median of 1,510 [1,100-4,601]. IgE levels were elevated in all patients, with a median of 799 [165-1,040] kU/L. Cases 1 and 3 had antinuclear antibodies. Immunophenotypic studies identified an abnormal T-cell clone in case 4. Clonal TCR-γ rearrangement was identified in case 5 but without aberrant cell surface immunophenotype.

#### ***Bronchoalveolar lavage fluid analysis***

Median BALF cellularity was 240,000 [160,000-390,000]/mm^3^. BALF eosinophilia was present in all cases, with a median of 28 [20-44] %.

#### ***Histological data***

Case 3 underwent transbronchial lung biopsies at diagnosis. Biopsies showed eosinophilic alveolo-interstitial infiltrates with alveolar septal thickening, eosinophils within the septa and alveoli filled with eosinophils and macrophages.

#### ***Pulmonary function tests***

Dynamic lung volume measurement was performed in four patients at admission. The two oldest patients had a combined ventilatory defect. The youngest had a decreased functional respiratory capacity indicating a possible restrictive pattern. The carbon monoxide diffusing capacity was measured in three patients and was abnormal for two of them.

#### ***Treatment and evolution***

All except one patient (case 4) received oral corticosteroids (Table 3). One patient (case 5) received, in addition to oral steroids, nine courses of high-dose-regimen intravenous corticosteroids (10 mg/kg/day methylprednisolone for 3 days per month). The mean initial dose of oral corticosteroids (prednisolone or prednisone) was 1.5 mg/kg/day (1-2 mg/kg/day). The duration of treatment was highly variable, with a median of 4 [3.5-6] months. Case 4 was treated with “low” doses of cyclosporine (5 mg/kg/day) and was still on maintenance therapy with this treatment after 39 months of follow-up.

**Table 3:** ICEP patient treatment and evolution

**Patient**	**Treatment**			**Evolution**						
	**Initial treatment**	**Duration (months)**	**Response**	**Total length of follow-up (years)**	**Relapse (number)**	**Delay after the end of the initial regimen (years)**	**Maintenance oral CS**	**Clinical evolution**	**Last CT scan**	**Last spirometry**
1	oral CS § 1 mg/kg/day	4	Total	1.5	0	_	_	_	m9: normal (1 nodule)	D
2	oral CS § 2 mg/kg/day	2	Total	5.9	0	_	_	asthma	m1: mild bronchiectasis	pO
3	oral CS * 1 mg/kg/day	4	Partial	8.1	2	0.8	0.07 mg/kg/day	asthma	y7: interstitial opacities and cysts	R, pO
4	cyclosporine 5 mg/kg/day	39	Total	3.2	0	_	_	asthma	y3: normal	mild R (obesity)
5	9 IV pulse + oral CS § 2 mg/kg/day	12	Partial	7.2	1	6.1	_	asthma	y6: interstitial opacities and cysts	normal

**Footnote:** § prednisolone, * prednisone, IV: intravenous methylprednisolone, CS: corticosteroid therapy, D: diffusion impairment, R: restriction, pO: peripheral obstruction ,m: month, y: year.

All patients presented a rapid clinical improvement, occuring within the first two weeks of treatment for 4 patients (cases 1, 2, 3, 4) and within the first month for the fifth (case 5.). Chest X-rays and HRCT scan normalized within one month for three patients and showed persistent interstitial opacities after the first month for two (cases 3 and 5) (Figures 1b and 2b).

The median length of follow-up was 5 years 2 months (range 1 year 5 months – 8 years 2 months). Cases 3 and 5 with persistent interstitial opacities relapsed clinically and biologically after weaning off initial corticosteroid treatment. These two children had a second regimen of oral corticosteroids and case 3 is still on maintenance oral corticosteroid therapy. The last spirometric follow-up showed a fixed restrictive and peripheral obstructive pattern in case 3 and was normal for case 5. The most recent HRCT scan showed thin-walled cyst development two and five years, respectively, post-episode (Figure 2b).

### IAEP

#### ***Epidemiology***

Three patients were identified (Table 4). There were two boys and one girl (sex ratio: 2). The median age at diagnosis was 14.08 [13.75-15] years. Two patients were current smokers and all had been exposed to environmental tobacco smoke at home for several years. Case 1 reported smoking cannabis for the first time 3 months ago and on a second occasion just a few hours before the onset of his respiratory symptoms. Case 3 reported an acute exposure to tear gas and a paint stripper containing a solvent mixture of DMSO and lactic acid.

**Table 4:** IAEP patient characteristics at diagnosis

**Patient**	**Clinical presentation**								**Biology**										**Imagery**	
	**Sex**	**Age at diagnosis (years)**	**Delay to diagnosis (days)**	**Atopic history**	**Active smoking**	**Passive smoking**	**Chronic exposure**	**Acute exposure**	**Initial blood Eo count/mm3**	**Maximal blood Eo count/mm3**	**Total IgE kU/L**	**Lymphocyte phenotype**	**ANA**	**BALF cellularity (cells/mm3)**	**BALF Eo ** **%**	**BALF lympho cytes %**	**BALF neutrophils %**	**BALF macro phages %**	**Chest X-rays**	**CT scan**
1	M	15.9	**4**	Yes	Yes	Yes	Dog, budgerigars	Cannabis	200	**3100**	nd	nd	_	350,000	**38**	16	18	28	AO, IO	**AO, GGO, IST, BVT, PE**
2	F	13.4	**6**	Yes	Yes	Yes	Dog, cat	No	nd	**680**	54	Normal	_	70,000	**26**	11	33	30	AO,IO,PE	**AO, PE**
3	M	14.1	**21**	No	No	Yes	No	Teargas, vironet	7800	**8500**	20.2	nd	_	1,200,000	**78**	7	7	8	AO, IO	**nd**

**Footnote:** F: female, M: male, nd: not determined, Eo: eosinophils, ANA: antinuclear antibodies, BALF: broncho-alveolar lavage fluid, AO: alveolar opacities, IO: interstitial opacities, PE: pleural effusion, GGO: ground-glass opacities, IST: interlobular septal thickening, BVT: peribronchiovascular thickening, in bold: diagnostic criteria.

#### ***Clinical and biological presentation***

The delay between the onset of symptoms and diagnosis was, respectively, 4, 6 and 21 days. All patients experienced exertional and, rapidly, rest dyspnea. A dry cough was present in only two patients. General symptoms appeared secondarily in < 48 hours or simultaneously with elevated fever.

#### ***Radiological data***

Chest X-rays showed bilateral dense infiltrates and ground-glass opacities in the three patients. One of the patients developed bilateral pleural effusion. A CT scan was performed for two patients and showed typical aspects of the adult form.

#### ***Bronchoalveolar lavage (BAL)***

The median BAL cellularity was 350,000 [210,000-775,000] /mm^3^. BAL eosinophilia was present in all cases, with a median of 38 [32-58] %.

#### ***Treatment and evolution***

All patients initially required ventilatory support with noninvasive ventilation for a few hours (2/3), invasive ventilation (3/3) with a median of 5 [3.5-7] days, and 4 days of extracorporeal membrane oxygenation (ECMO) for case 1 (Table 5). High-dose intravenous corticosteroids were initiated (4, 6 and 10 mg/kg/day methylprednisolone, respectively) with a progressive decrease and shift towards the oral route for a total length of 90, 48 and 42 days, respectively. This treatment resulted in a dramatic clinical improvement with rapid weaning of ventilator support. Chest X-ray controls at 6 and 11 days were normal. The CT scan was considered normal at one month for case 2 and at 8 months for case 3. Spirometry performed at two months was normal for cases 1 and 3, but showed a decreased carbon monoxide transfer factor and transfer coefficient in case 2. None of the patients relapsed.

**Table 5:** IAEP patient treatment and evolution

**Patient**	**Treatment**								**Evolution**			
	**Ventilatory support**				**Corticosteroid treatment**				**Total length of follow up**	**Asthma**	**Last spirometry**	**Last CT scan**
	**NIV**	**MV**	**ECMO**	**paO2/FiO2**	**IVCS**	**OCS start dose**	**Total length**	**Response**				
1	_	5 days	_	77	4 mg/kg/day	prednisolone 2 mg/kg/day	3 months	Total	3 years	Yes	Normal	nd
									10 months			
							1 week					
					7 days							
2	few hours	9 days	4 days	45	6-4 mg/kg/day	prednisone 2 mg/kg/day	48 days	Total	9 months	No	D	Normal
					9 days							
3	24 h before, 48 h after MV	2 days	_	50	10 mg/kg/day	prednisolone 1 mg/kg/day	6 weeks	Total	8 months	No	Normal (DLCO not done)	Normal
					3 days							

**Footnote:** NIV: noninvasive ventilation, MV: mechanical ventilation, ECMO: ExtraCorporeal Membrane Oxygenation, IVCS: intravenous corticosteroid therapy, OCS: oral corticosteroid therapy, D: diffusion impairment.

## Discussion

We present the first series of pediatric ICEP and IAEP diagnosed in a 5-year time frame in the same network of French Rare Lung Diseases.

### ICEP

Described by Carrington et al. [5], ICEP is a rare disease accounting for < 3% of interstitial diseases, but it is the major cause of eosinophilic lung disease in countries with a low prevalence of parasitic infections. It predominates in females (sex ratio 0.5) with a mean age at diagnosis of 45 years, and less than 6% of patients are less than 20 years old [3]. More than 50% of cases have a prior history of atopy and asthma [6]. Patients are usually nonsmokers. ICEP has a progressive onset with development of respiratory (mild dyspnea, cough) and systemic (fatigue, malaise, fever, anorexia, night sweats and weight loss) symptoms over several weeks. The imaging features are rather characteristic with, in almost all cases, bilateral peripheral alveolar infiltrates with ill-defined margins on the chest radiograph and coexisting peripheral ground-glass on CT scan [4]. The blood eosinophil count is elevated in 90% of cases. BAL eosinophilia is now a major diagnostic procedure in ICEP: it is always present, commonly at around 40% in adults. ICEP responds dramatically, clinically, biologically and radiologically, to oral corticosteroids in adults and a short regimen of 6 weeks is now used. Relapses are frequent (> 50%) and require a longer regimen with low-dose corticosteroid therapy [7].

ICEP is extremely rare in children. To date, only ten pediatric observations have been reported in the literature [8-14], apart from those published by our center [15-17], with no follow-up studies or series (Table 6). Diagnosis in children has relied on adult criteria [3], as used in this study. Lung biopsy, no longer required for diagnosis, shows eosinophilic infiltration of the lung structures.

**Table 6:** Data from the literature on pediatric ICEP

**Year**	**First Author**	**Journal**	**Sex**	**Age at diagnosis (years)**	**Asthma history**	**Fever**	**Blood Eo count/mm3**	**BALF**	**Histology**	**Total length treatment (months)**	**Relapse**	**Chest X-rays**	**CT scan**
1975	Rao M	Chest	M	1	No	Yes	Elevated	No	Yes	12	No	AO, Ad	**nd**
1992	Naughton M	Chest	F	15	Yes	No	3200	**nd**	No	9	Multiple	AO	**nd**
1993	O’ Sullivan BP	J Pediatr	M	14.9	Yes	Yes	3350	No	Yes	**nd**	**nd**	AO	AO, BBO
2000	Oermann C	J Pediatr	F	16.1	No	No	4030	No	Yes	**nd**	No	AO, IO	**nd**
2003	Wubbel C	Chest	F	6-10	**nd**	**nd**	**nd**	**nd**	**nd**	**nd**	**nd**	**nd**	**nd**
2003	Wubbel C	Chest	F	6-10	**nd**	**nd**	**nd**	**nd**	**nd**	**nd**	**nd**	**nd**	**nd**
2003	Wubbel C	Chest	M	11-16	Yes	Yes	9504	Yes	Yes	**nd**	**nd**	**nd**	AO
2003	Wubbel C	Chest	M	11-16	**nd**	**nd**	**nd**	**nd**	**nd**	**nd**	**nd**	**nd**	**nd**
2005	Tanir G	TuberkToraks	M	4	Yes	**nd**	2626	Yes	Yes	**nd**	**nd**	**nd**	AO, GGO
2010	Cakir E	Pediatr Pulmonol	F	7	Yes	No	2205	Yes	No	6	No	**nd**	AO, GGO

**Footnote:** F: female, M: male, **nd**: not determined, Eo: eosinophils, BALF: broncho-alveolar lavage fluid, AO: alveolar opacity, IO: interstitial opacities, Ad: adenopathy, BBO: basal band of opacities, GGO: ground-glass opacities.

Our study has collected comprehensive data for all the pediatric cases diagnosed and allows some comparisons to be made with the typical adult form [7], even though the number of subjects is limited. Only two patients out of five had alveolar opacities and three had an interstitial aspect with ground-glass opacities and micronodular syndrome and for two of them a persistence of initial elements, with the late appearance of thin-walled cysts for both. We can thus distinguish two main subsets of patients.

The first subset (cases 1, 2 and 4) had a dramatic clinical, radiological and biological improvement after the first line of treatment, with no relapse to date. Cases 1 and 2 had a similar form to adults, and recovered fully after initial oral corticosteroid treatment. Case 4 had a 2.2% abnormal T-lymphocyte (TL) clone and a respiratory-only presentation with “patchy-only-interstitial” HRCT scan. The distinction between lymphoid-variant hypereosinophilic syndrome (L-HES) [18] and ICEP was questionable for this case. Indeed lung involvement in HES consists mainly of patchy ground-glass opacities as presented by our patient [19]. However, we considered that case 4 had ICEP, considering the respiratory-only setting (single-organ disease) without any other organ involvement at diagnosis and during follow-up (clinical follow-up and cardiac ultrasound performed at 1 month, 6 months and then yearly) [18] and the low level of the abnormal TL clone in comparison with levels reported in L-HES [20] – this clone having been described at low levels in the general population [21]. Moreover, Cottin et al. recently reported T-cell abnormalities in eosinophilic lung disease, showing that the pathophysiologic boundary with HES may be very thin (1/9 adult patients with ICEP had T-cell receptor gamma rearrangements and 1/57 adults with eosinophilic lung disease had an aberrant T-cell population immunophenotype) [22].

The other subset (cases 3 and 5) had a persistent interstitial form with the appearance of thin-walled cysts for both after more than five years of stability of the initial lesions for case 5 and two years of follow-up for case 3. They, moreover, had very limited general symptoms and less peripheral blood hypereosinophilia as well as lower BAL eosinophilia compared to the adults. One patient had, at diagnosis, a biopsy showing an initially typical histological aspect of ICEP. The other patient had a T-cell clonality, as recently described in ICEP [22]. Their clinical and radiological follow-up might, nevertheless, suggest revisiting the final diagnosis. Indeed, mild BALF eosinophilia has been described in adult idiopathic pulmonary fibrosis (IPF), in up to 50% of patients with desquamative interstitial pneumonia (DIP), in cryptogenic organizing pneumonias and nonspecific interstitial pneumonitis [23-27]. Peripheral blood eosinophilia is rarely found in UIP and DIP (around 10% of cases) [27]. ICEP can evolve, moreover, spontaneously into lung fibrosis with a DIP-like reaction [28]. All of these data indicate that there is still a place for lung biopsy in the diagnosis of ICEP when the initial setting and evolution are not usual. The modality of these biopsies also needs to be clarified, as case 3, with an unusual evolution, was initially described as having a standard histopahologic finding of ICEP on transbronchial biopsies. In cases not responding to initial oral corticosteroid treatment, diagnostic biopsies should be performed after a period of weaning off treatment, to contribute to the differential diagnosis between ICEP and idiopathic interstitial pneumonia. However, the optimal length and the feasibility of this weaning period need to be established.

The frequency of unusual forms in this first pediatric national series suggests the specification of a more strict pediatric definition, including the definition of the requirement for biopsy. The treatment regimens of these patients should be modified and the use of corticosteroid-sparing agents in the context of an eosinophil-triggered disease evaluated (omalizumab, mepolizumab) [29]. Low-dose cyclosporine, used by other teams in lymphoid-variant HES and idiopathic HES [30], should be further evaluated in the context of eosinophilic pneumonias [31], and especially in persistent forms of ICEP.

Regarding hypereosinophilic asthma, the boundary [32] can be very difficult to specify in young children, and two patients who were excluded from our cohort were finally classified as asthmatic. Hypereosinophilic asthma can be observed in pediatric patients with > 25% eosinophils in BALF. Asthma attacks are induced by viral infections in 80% of cases [33] and alveolar opacities and atelectasis are frequent. Limits can be difficult to establish initially as more than 50% of ICEP patients have previously been diagnosed as asthmatic [7]. Only the clinical evolution of the patient can help the evaluation of the clinician.

In order to consider these pediatric specificities, the definition of ICEP could be clarified in children as:

i) diffuse pulmonary alveolar consolidation with air bronchogram and/or ground-glass opacities, especially with peripheral predominance;

ii) BALF eosinophilia > 20% or peripheral blood eosinophilia > 1×10^9^ cells/L;

iii) respiratory symptoms present for more than 4 weeks;

iv) absence of other known causes of eosinophilic lung disease;

v) consistent open lung biopsy for cases without initial dramatic clinical AND radiological improvement on first-line treatment.

Two items of adult classification concerning imaging and the exclusion of differential diagnoses were not changed. Clinical and biological aspects were adapted in order to be fulfilled by most of the pediatric cases. As discussed previously, the requirement for open lung biopsy has been specified in cases of resistance to first-line treatment.

**Figure 3:**
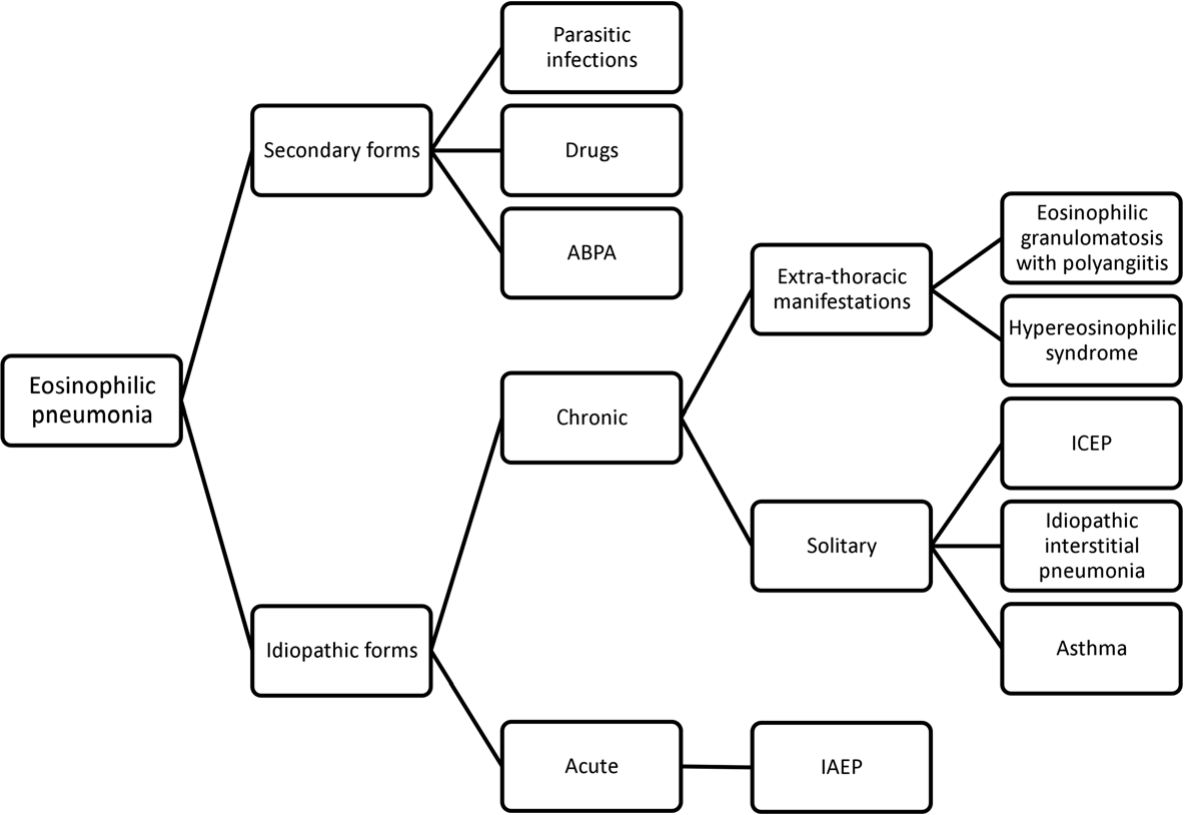
Classification algorithm in pediatric eosinophilic pneumonias.

A new classification algorithm for eosinophilic pneumonia in children is also proposed (Figure 3), including two main differential diagnoses to ICEP: hypereosinophilic asthma and idiopathic interstitial pneumonia. The overall impression is that idiopathic chronic eosinophilic pneumonia is part of the clinical spectrum of a wider group of lung diseases, with some potential overlap between them, making a clear distinction sometimes difficult. Molecular analysis should likely help in the clarification of this aspect and better circumvent underlying biological processes.

### IAEP

We describe three cases of IAEP in adolescents. Specific descriptions of pediatric IAEP are rare [34-36], but such patients have also been included in series mixed with adults [37]. To date, no specific pediatric series have been described but pediatric cases are often adolescents. Acute exposure to irritants was found in our series (smoke, cannabis, paint stripper and tear gas), as in adults. The severity of respiratory distress requiring ECMO 24 hours after hospitalization and 48 hours after the beginning of symptoms has never been described in adults. This does not correspond to a delay in medical care but rather to the rapid development of an aggressive pathology. Other clinical, biological and radiological aspects of these patients seem similar to those in adults. The length of steroid treatment was highly variable, as in adults, and should be reduced to two weeks [38]. Treatment resulted, as in adults, in a dramatic clinical improvement, with rapid weaning of ventilator support. None of the patients relapsed. CT scan control was normal and initial spirometric follow-up showed only minor alterations.

## Conclusion

ICEP in children, even respecting the definition established by adults, seems to be polymorphous. We propose here a specific pediatric definition of ICEP and we argue for an open lung biopsy proof of the disease in persistent interstitial forms. As in adults, oral steroids seem to be an efficient treatment in children, and the duration could probably be shortened in classical forms. New therapeutic options in ICEP (mepolizumab, omalizumab,…) could be interesting in order to spare oral corticosteroids. IAEP seems to be similar in its initial presentation to adult forms in our series but its short-term course may be more severe, nevertheless with a good prognosis. This study represents the largest pediatric cohort ever described in the literature, but is still based on a small number of patients. These pediatric orphan diseases should be registered internationally in order to improve our knowledge, especially in chronic forms.

## Competing interest

All authors declare that they have no competing interest.

## Authors’ contributions

LGC, AC and JdB designed the study and drafted the manuscript. All the authors contributed to acquisition of the data notably through Respirare® database. AH, NN, MF, TL, AC and JdB made a special contribution to manuscript corrections. All the authors read and approved the final manuscript.
